# The Role of Tourism and Recreation in the Spread of Non-Native Species: A Systematic Review and Meta-Analysis

**DOI:** 10.1371/journal.pone.0140833

**Published:** 2015-10-20

**Authors:** Lucy G. Anderson, Steve Rocliffe, Neal R. Haddaway, Alison M. Dunn

**Affiliations:** 1 School of Biology, University of Leeds, Leeds, United Kingdom; 2 Centre for Environment, Fisheries and Aquaculture Science, Weymouth, United Kingdom; 3 Environment Department, University of York, York, United Kingdom; 4 MISTRA EviEM, Royal Swedish Academy of Sciences, Stockholm, Sweden; Bournemouth University, UNITED KINGDOM

## Abstract

Managing the pathways by which non-native species are introduced and spread is considered the most effective way of preventing species invasions. Tourism and outdoor recreation involve the frequent congregation of people, vehicles and vessels from geographically diverse areas. They are therefore perceived to be major pathways for the movement of non-native species, and ones that will become increasingly important with the continued growth of these sectors. However, a global assessment of the relationship between tourism activities and the introduction of non-native species–particularly in freshwater and marine environments–is lacking. We conducted a systematic review and meta-analysis to determine the impact of tourism and outdoor recreation on non-native species in terrestrial, marine and freshwater environments. Our results provide quantitative evidence that the abundance and richness of non-native species are significantly higher in sites where tourist activities take place than in control sites. The pattern was consistent across terrestrial, freshwater and marine environments; across a variety of vectors (e.g. horses, hikers, yachts); and across a range of taxonomic groups. These results highlight the need for widespread biosecurity interventions to prevent the inadvertent introduction of invasive non-native species (INNS) as the tourism and outdoor recreation sectors grow.

## Introduction

Understanding and managing the pathways by which non-native species are introduced into new regions is considered the most effective way to prevent future biological invasions [1–6]. As such, effective pathway management forms one of the Convention on Biological Diversity’s Aichi Biodiversity Targets for 2020 and is a key element of the new EU regulation 1143/2014 on the prevention and management of the introduction and spread of INNS [7,8]. However, the development of pathway management plans and biosecurity measures must be grounded in evidence about the vectors and mechanisms by which non-native species can be transported [4,5,9].

Tourism is considered to be a major pathway for the spread of non-native species [10–13]. Not only can the congregation of large numbers of people, vehicles and vessels from geographically diverse areas provide a regular supply of non-native propagules [14,15], common recreational activities such as hiking, mountain biking and off-road driving can act as forms of habitat disturbance, potentially facilitating species invasion [14,16–18]. Disturbance occurs when an activity either partially or totally destroys the plant/animal biomass in an area, changing niche opportunities for the species within the habitat [16,19]. Non-native species are often particularly successful in disturbed habitats as their superior rates of growth and reproduction enable them to quickly colonise disturbed areas [16,20,21].

Existing research has focused on the role of tourism and outdoor recreation as vectors for non-native species in terrestrial environments, notably protected areas and national parks (for example [18,20,22–24]. There, as transport vectors are often restricted, recreational activities form one of the few pathways by which non-native species can be introduced [18]. Previous studies have revealed that activities such as hiking and horse-riding can act as vectors for the dispersal of non-native seeds as well as pathogens such as *Phytophthora ramorum*, the causative agent of sudden oak death [22,25,26].

Despite the terrestrial focus in the literature to date, recreational activities can also act as vectors for the introduction of non-native species in aquatic environments [27–29]. For example, recreational boats have been a major vector for the spread of the zebra mussel *Dreissena polymorpha* and invasive non-native macrophtyes between lakes and rivers within Europe, the USA and New Zealand [30,31]. In marine environments, yachts have been responsible for introducing non-native bivalves, algae, ascidians and bryozoan into ports in Australasia and the Caribbean [32–34]. Yet to date, there has been no quantitative global review of the impacts of tourism and recreation on the abundance and richness of non-native species in aquatic systems.

Internationally, tourist arrivals are expected to grow from 1 billion in 2013, to 1.8 billion by 2030 [35] and nature-based tourism (i.e. wildlife viewing and outdoor recreation, often centred around protected areas and national parks) is a key growth area [18,36–38]. As nature-based tourism and outdoor recreation (hereafter grouped under ‘recreation’ for simplicity) often take place in relatively pristine habitats, biodiversity hotspots and in developing countries which rely upon tourist income [37], it is vital to better understand the invasion pathway represented by tourist activities, so that it can be effectively managed.

Meta-analysis provides a valuable tool with which to quantitatively synthesize the results of multiple studies to identify large scale patterns and facilitate evidence-based conservation management [39,40]. The aim of this study was to conduct a systematic review and meta-analysis to quantitatively determine whether the richness and abundance of non-native species were higher in sites where recreation took place than sites where it did not in terrestrial, freshwater and marine environments.

## Materials and Methods

### Search Strategy

We performed our literature review following recognised protocols for systematic reviews and meta-analyses [41,42].

On 15 March 2014 we searched for relevant studies using three academic literature databases: Scopus, ISI Web of Science (University of Leeds database subscriptions detailed in Supplementary Information) and Science Direct.

The following Boolean search string was used in each database: ("horse riding" OR "mountain biking" OR "bicycle" OR "cyclist" OR "off-road vehicle" OR "4x4 vehicle" OR "all-terrain vehicle" OR "rock climbing" OR mountaineer* OR "scuba div*" OR surf* OR angl* OR boat* OR vessel OR anchor OR canoe OR kayak* OR sail* OR yacht* OR "leisure craft" OR "personal water craft" OR "cruise ship" OR "passenger ship" OR ferry OR camp* OR hik* OR trails OR "walking tracks" OR paths OR safari OR ski* OR snowboard* OR wintersport OR "wildlife watch*" OR “bird watch*” OR visitor OR touris* OR ecotour* OR eco-tour* OR passenger OR travel* OR leisure OR sightsee* OR footwear OR luggage OR clothing OR "tourist transport" OR "tourist vehicle" OR train OR railway OR car OR vehicle OR coach OR bus OR recreation OR aeroplane OR "air transport" OR airport OR plane OR "human vector" OR "human activity" OR (("protected areas" OR "nature reserve" OR "national park" OR "marine reserve" OR “marine park” OR "marine protected area") ***AND*** *(visitor OR user OR tourist))) AND ("invasive species" OR "introduced species" OR "non-native species" OR "alien species" OR "non-indigenous species" OR "exotic species")* ***AND*** *("species richness" OR diversity OR cover OR abundance OR density OR biomass)*.

The first 100 hits of an advanced search performed using Google.com were also checked for relevance. Our search was restricted to English language search terms but included all publication years. The list of tourist and recreational activities was collated from previous studies of tourism in terrestrial, freshwater and marine environments [28,43,44]. Not all studies differentiated between invasive and non-invasive non-native species. Moreover, studies used different terms and definitions to categorize invasive and non-native species (an issue affecting in the INNS literature at large [45]). Because of this, we treated non-native species as one group, irrespective of whether they were invasive.

### Screening and data-extraction

**Fig 1:**
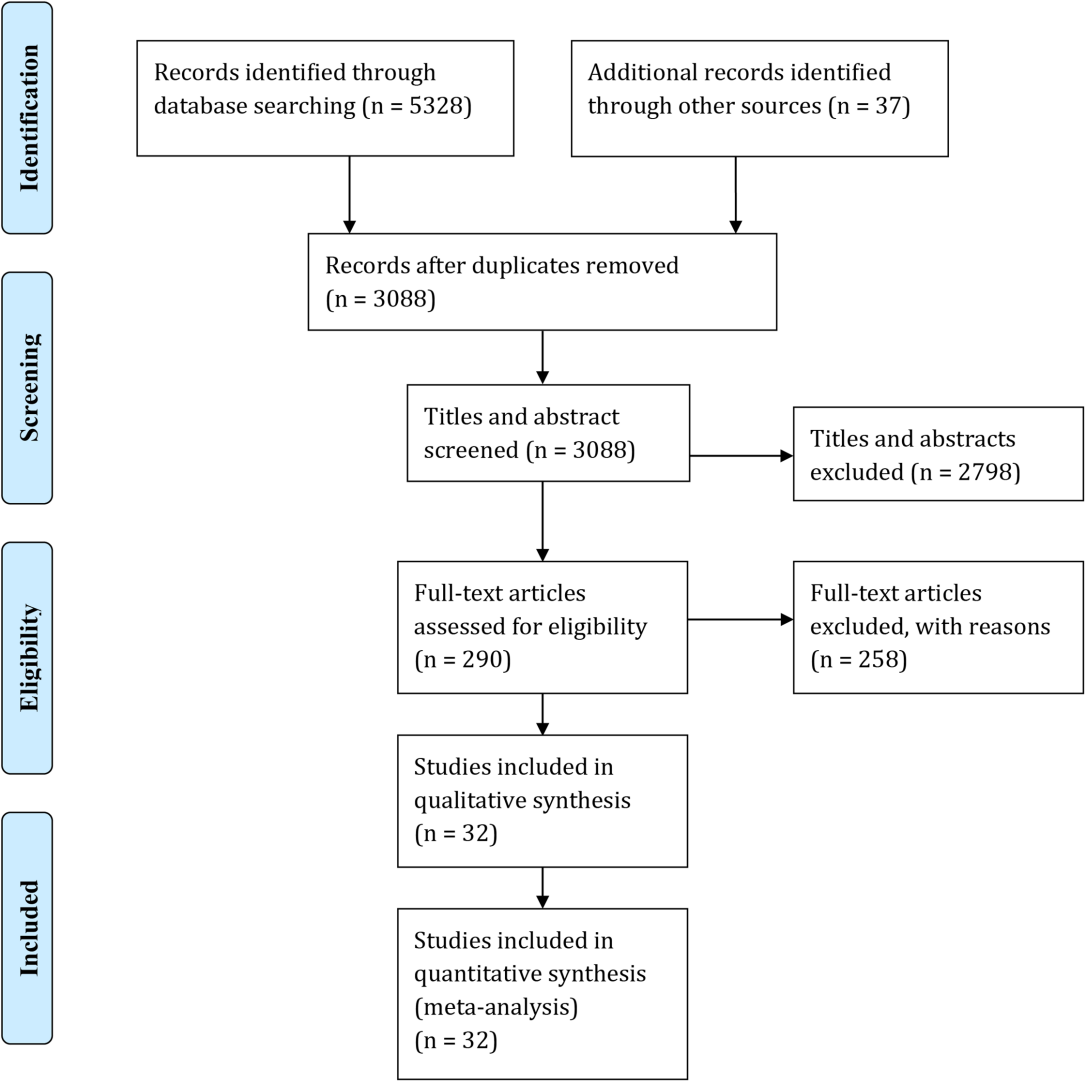
PRISMA Literature Search Flow diagram. Diagram depicts the number of studies retained and discarded at each stage of the literature search.

Our original search returned 3088 studies after duplicates were removed. Titles and abstracts were vetted by two reviewers (LA and SR) and the Kappa statistic [46] was used to evaluate inter-reviewer agreement (Kappa = 0.84: near perfect level of agreement) (Fig 1).

We retrieved and reviewed 290 full text articles against inclusion and critical appraisal criteria (Table 1). Includible studies could be observational or experimental in nature but had to have the primary goal of quantifying the impact of a tourist or outdoor recreation activity, tourist-specific transport vector or visitors to a tourist destination (such as a national park or island). After full text screening, quantitative data were extracted from 32 studies and taken on to meta-analysis. This set of studies represented 37 effect size data points (species richness n = 18, abundance n = 19, Fig 1). Full details of the included studies are summarised in S1 Table.

**Table 1:** Criteria for study inclusion and critical appraisal during quantitative synthesis. Studies excluded at the full text stage are available in supplementary material.

Inclusion criteria	Critical appraisal criteria
1. Primary study including a quantitative comparison of abundance (e.g. biomass, density, percentage cover, total abundance) and/or species richness (total number of species, mean number of species, proportion of species or Simpson’s diversity index) of non-native species in a site affected by a recreational activity, and a comparable control site, or at the same site before and after recreational activity began.	1. Experiment not replicated (only one treatment and one control site).
2. Study provides exact P value or a statistical result (Z, F, t, r, r^2^ or X^2^) accompanied by the sample size or degrees of freedom. Alternatively, study can provide raw data on the mean abundance/species richness in the treatment and control sites or at the same site before/after the intervention with associated sample sizes.	2. No control site, or insufficient information provided about the characteristics of the control site to assess its suitability.
	3. Study does not report confidence intervals or sample sizes.
	4. Treatment and control sites spatially confounded.
	5. Study includes evidence of intentional non-native species introduction which may confound results. For example through seeding (ski-resorts) or stocking (angling lakes).
	6. Study of road/vehicles, railways or boats where it is unclear whether the primary vehicles/vessels are industrial (e.g. cargo ships, goods trains, works vehicles) or strictly tourist related (yachts, recreational boats, tourist cruise ships etc.)

We coded each study according to sample size, sample selection (purposive, randomised, blocked, not stated, other) spatial scale (<2 ha; 2-10ha; >10ha), temporal scale (<2 year since tourist activity began; 2–5 years or >5 years), the activity/vector in focus, habitat type (freshwater/marine/terrestrial), study taxa, study design (Before-After (BA); Control-Impact (CI); Before-After-Control-Impact (BACI); other), whether the study was observational or experimental and data on abundance (biomass, density, percentage cover, total abundance) and species richness (total number of species, mean number of species, proportion of species or Simpson’s diversity index). For studies where abundance/species richness data were separated across spatial or temporal scales, we selected the mean value, weighted by the sample size at each spatial/temporal scale [47]. Where results were presented graphically, we extracted the mean and variation (e.g. standard error or 95% confidence intervals) from the figure using *ImageJ* [48]. To avoid double-counting, each study could only contribute one abundance and/or one richness effect size to the meta-analysis.

### Effect size

For each study, we calculated the effect size as the difference between the abundance/species richness of non-native species in control sites and sites experiencing recreational activity using the R package *compute*.*es* [49]. A value of 0.001 was added to raw abundance and species richness figures in order to calculate the effect size of studies where non-native species were not found in the control site [50]. We used *Hedges g* as a weighted and standardised effect size metric [51]. Positive *g* values indicate that non-native species richness or abundance was higher in sites with tourist activity than in undisturbed sites. A value of *g* greater than or equal to 0.8 can be interpreted as a large effect size; 0.5 a moderate effect size; and 0.2 is a small effect size [52].

### Full models

Using the *metafor* package in R [53,54], we created random effects models to calculate the grand-mean effect size across all non-native species abundance studies, and all non-native species richness studies. Random effects models are considered appropriate for ecological studies because they allow effect size estimates to vary both due to sampling error and as a result of real ecological differences between studies [55]. Due to the small sample size of our studies, we calculated bias-corrected 95% confidence intervals around the two mean effect sizes by bootstrapping 10,000 iterations using the *boot* package in R [56]. The grand mean effect size was considered to be significantly different from zero if the confidence intervals did not overlap zero.

### Factors explaining heterogeneity in effect sizes

The total heterogeneity statistic (Q) was used to determine whether the heterogeneity in grand mean effect sizes was significantly greater than what would be expected from sampling error alone [41,51]. Where the Q statistic was significant, sub-group analyses were conducted using mixed effects models with moderators (study ID included as a random factor) to determine whether ecosystem (terrestrial/aquatic), taxa, study type (observational/experimental) or vector type could explain the variation in effect sizes. Parametric 95% confidence intervals (suitable for sample sizes of n<10 [55]) were calculated around each subgroup mean to determine whether the mean effect size had a significant effect on non-native species richness/abundance.

### Assessment of publication bias

We used a number of standard methods to check for publication bias in our analyses. A visual assessment of effect size plotted against the standard normal distribution (normal quantile plot) revealed that all data points fell within 95% confidence intervals (S1 Fig). Failsafe tests revealed that it would take an additional 864 abundance studies and additional 1415 species richness studies with effect sizes of zero to change the result of our analysis from significant to non-significant [57]. Finally, rank correlation tests were non-significant for abundance (Kendall’s tau = 0.09, p = 0.62) and species richness (Kendall’s tau = 0.05, p = 0.79) indicating that there were no significant correlations between effect size and variance. We are therefore confident that our meta-analyses are highly unlikely to be affected by publication bias.

## Results

The 32 studies included in our systematic review had a broad geographic distribution, however the majority were from North America (n = 13), Australasia (n = 6) and Europe (n = 4) (Full details in S1 Table). They comprised 22 terrestrial studies, eight marine studies and two freshwater studies. Due to the small sample size of freshwater studies (n = 2), we combined freshwater and marine studies into an “aquatic” category (n = 10). Six key recreational activities (interventions) were identified from the studies included in our analysis. They included: visits to national parks, hiking, horse-riding, recreational boating, yachting and the recreational harvesting of shellfish.

**Fig 2:**
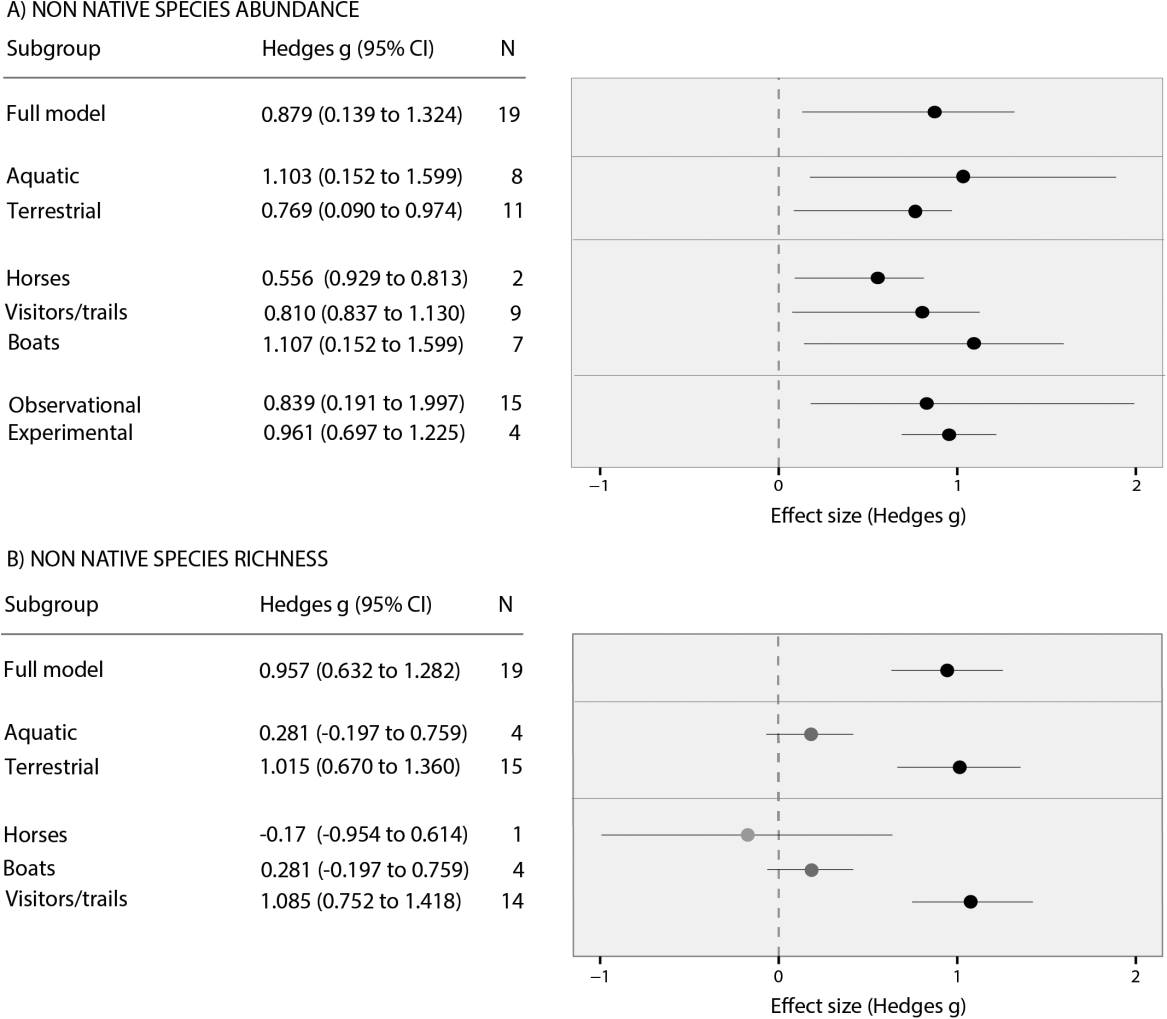
Meta-analysis forest plots. Plots show the effect of recreational activities on A) non-native species abundance and B) non-native species richness. Effect size values >0 show that the species richness or abundance of non-native species was greater in sites where recreational activities took place. The mean effect size and 95% confidence interval is shown for the overall result and each sub-group analysis. Bias-corrected confidence intervals were bootstrapped for groups n<10 and parametric confidence intervals for groups n≥10. Confidence intervals that overlap the dashed line at zero are not significantly different from zero.

The large positive effect sizes obtained from our meta-analyses indicate that both the abundance (mean effect size (g) = 0.89, p<0.001) and species richness (mean effect size (g) = 0.96, p<0.001) of non-native species were significantly higher in sites that where tourist activities took place than in control sites (Fig 2). The pattern was repeated in both terrestrial and aquatic environments, across multiple non-native taxa (including terrestrial and aquatic plants, invertebrates and fungal pathogens) and a suite of tourist-related vectors (Fig 2).

In both our non-native species richness and abundance meta-analyses, the Q statistic was significant (Table 2), indicating that the heterogeneity in effect sizes between studies was higher than would be expected by sampling error alone [41,55]. However**,** none of our subgroups (vector type, habitat type, study type (observational/experimental) or aquatic vs. terrestrial) were significant predictors of between-group heterogeneity (Table 2), suggesting that the impact of tourism was similar across all vectors/habitats. Due to high levels of correlation between study type (observational/experimental) and spatial scale; and between study type and temporal scale, we only included the subgroup study type in our analyses.

**Table 2:** Total heterogeneity (Q_T_) and between-group heterogeneity (Q_B_) of effect sizes in studies comparing the abundance and richness of non-native species between sites where recreational activities took place vs. control sites. As there was a significant correlation between study type, study area and duration of study, only study type was analysed in the subgroup analysis.

Explanatory variable	Non-native abundance				Non-native species richness			
	QT	QB	df	*p*	QT	QB	df	*p*
	98.81		17	<0.0001	88.97		18	<0.0001
Vector type		1.82	3	0.71		4,52	2	0.10
Aquatic vs. terrestrial		1.45	1	0.22		1.45	2	0.48
Ecoregion (Ter/Mar/Fw)		2.94	2	0.23		1.13	2	0.28
Study type		0.44	1	0.50		NA (only observational studies)		

Forest plots of the mean effect size and bootstrapped confidence intervals for each subgroup suggested that both terrestrial and aquatic recreational activities had a large and significant positive effect on the abundance of non-native species (Fig 2A). No significant differences in effect size were detected between vectors in our analyses of non-native species abundance, however boats appeared to have a larger effect on non-native species abundance than horse riding and hiking (Fig 2A).

In contrast, when the results were broken down by vector in our analyses of non-native species richness, only terrestrial activities (visitors to national parks/trails) had significant positive effects on non-native species richness (Fig 2B). Although boats appeared to have a positive effect on non-native species richness, this was not significant (Fig 2B). The only study investigating the impact of horse-riding on non-native species richness did not show a significant effect, however the small sample size was too small to draw a firm conclusion.

## Discussion

Effective INNS management requires an understanding of the relative importance of different pathways of spread [5]. Our results provide quantitative evidence in support of the hypothesis that tourism is a pathway for the spread of non-native species across the globe [10,12,13]. The results of our meta-analysis demonstrate that the abundance and richness of non-native species are significantly higher in sites where recreational activities took place than in control sites, and that this pattern is consistent across multiple non-native taxa, in both terrestrial and aquatic habitats, and across a suite of different vectors.

The literature search revealed that the majority of empirical studies conducted to investigate the impacts of recreational activities on INNS are terrestrial in focus (15/18 studies of non-native species richness and 11/19 studies of non-native species abundance). The meta-analysis revealed that there was a significantly higher abundance and richness of non-native species in terrestrial sites with recreational activities than control sites (Fig 2). These results were in accord with previous studies. For example, a review of 18 vegetation surveys in Kosciuszko National Park, Australia, revealed that 48 non-native species had been reported in the park’s natural vegetation compared to 152 in areas where tourist activities took place [18]. In addition, a long term study of visitors to US National Parks showed that there were significantly higher numbers of non-native species in parks with higher visitor numbers [22], a pattern that was reflected in forests with/without visitor access in Poland [58]. However, unlike many previous studies this meta-analysis incorporated tropical and temperate habitats and both continental and island studies.

Although only two studies could be included, horse-riding did appear to have a significant effect on the abundance of non-native species, with significantly more reported in sites where horse-riding took place, than in control sites. We believe further control-impact studies are required to fully understand the impact of this vector.

Unlike the aquatic studies included in our meta-analysis that incorporated a range of non-native plants, algae and invertebrates, the majority of terrestrial studies (21/22) focused on non-native plants. The only non-plant terrestrial study [25] showed that the prevalence of the fungal pathogen *Phytophthora ramorum* was higher on trails than in undisturbed vegetation in a Californian National Park. The impact that terrestrial recreational activities are having on other types of non-native taxa (such as other pathogens and invertebrates) demands further attention.

Our study is the first quantitative global analysis of the relationship between recreational activities and non-native species in marine and freshwater environments. In accord with findings on terrestrial activities, our meta-analysis revealed that the abundance of aquatic non-native species–including seagrasses, seaweeds, macrophtyes, molluscs, amphipods and bryozoans–were significantly higher in aquatic environments where recreational boating or yachting took place, than in control sites. No significant differences in effect size were found between terrestrial and aquatic environments, suggesting that the impacts of recreational activities are similarly important, and require management interventions of a similar magnitude.

Recreational boating and angling are receiving growing recognition as vectors for non-native species [31,59–61] and are thought to have been responsible for over a third of non-native species introductions into Europe [62]. Examples include the introduction of the zebra mussel *Dreissena polymorpha* from England to Ireland via the hulls of recreational boats [30] with subsequent impacts on the fisheries, water treatment works and aquatic transport industries [63]; and the introduction of the Ponto-Caspian gammarid shrimp *Dikerogammarus villosus* into watersports lakes in the UK and European Alps [60,64]. In New Zealand, the distribution of the invasive non-native diatom didymo (*Didymosphenia geminata*) is consistent with angler-mediated introduction and dispersal [65]. The invasion was first reported in 2004 and had cost the New Zealand government over NZD $127.8 million to manage by 2011 [66].

In marine environments, the introduction of non-native species through aquatic activities could potentially compromise the conservation value of marine reserves. Much like their terrestrial counterparts, marine reserves can attract high rates of visitation by tourists, leading to a congregation of potential transport vectors including boat anchors, SCUBA equipment, boat ballast and bilge water and fouled hulls [67]. The reduced levels of harvesting within marine reserves may also ironically allow non-native species that are inadvertently introduced to become more abundant [68], however others argue that marine reserves are associated with greater native species richness [69] and are therefore more resilient to biological invasions [70].

In addition to marine reserves, long distance yachts could be one of few vectors capable of introducing INNS to the marine environment surrounding oceanic islands [32], ecosystems in which species invasions are considered the most acute threat to biodiversity loss [6,71]. Yachts are thought to be responsible for the introduction of five non-native species including sponges, a macroalga, a bryozoan and a hydroid to Palmyra, an unoccupied North Pacific atoll [72] as well as introducing *Halophila stipulacea*, a non-native seagrass, to many islands across the Caribbean [34].

### The relationship between study design and effect size

Forest plots revealed that the mean effect of tourism on non-native species abundance was higher in experimental studies than observational studies, although both were significant. In our study, experimental studies occurred at smaller spatial and temporal scales than observational studies, typically < 1ha in area and <1 year in duration. The larger effect size of experimental studies may be explained by experimental simulations being more intense or happening more suddenly than would take place in natural conditions, whereas effects of non-native species in observational studies may have been diluted by space and time [21].

### Additional impacts of tourism

We focused our study on tourist activities associated with unintentional non-native species spread. However, tourist infrastructure–for example the building of footpaths and lodges, and the planting of non-native species in hotel gardens and ski resorts–have also been associated with the intentional introduction of non-native species. For example, tourist development was identified as the main determinant of non-native plant abundance and richness in study of 37 Mediterranean Islands [73]. Similarly, 152 of the 156 non-native plants recorded in Kosciusko National Park in Australia were associated with tourist infrastructure including ski resorts and hotel gardens [18], and the seeding of ski runs in the conversion of alpine habitats to ski resorts is also a source of non-native species spread to neighbouring areas [74,75]. A review of the links between tourist infrastructure and the abundance/richness of non-native species would add valuable further insight into the link between tourism and non-native species.

### Issues relating to study design

A major obstacle encountered during this study was the paucity of studies that provided sufficient information to calculate an effect size. For example, 24% of the studies that reached the final stage of assessment (n = 69) failed to meet the inclusion criteria based on the lack of a control site in their experimental design. Not only did this lead to an unintentional dominance of plant-based studies in the meta-analyses, it revealed a wider issue in ecological study design. As research techniques in applied ecology begin to follow the rigorous systematic methods which have been adopted in healthcare science, we implore ecologists to adopt balanced study designs from which effect sizes can be calculated [39,76–78]. This will facilitate the use of meta-analysis in ecology as an evidence-based conservation management tool [39,40].

### Management implications

As nature-based tourism continues to grow in popularity [36], the tourist-assisted transport of non-native species to remote habitats such as oceanic islands, polar regions (previously considered so remote they were ‘immune’ to invasions [6]) and biodiversity hotspots, could have catastrophic consequences. This is because the endemic flora and fauna living in these environments have often evolved in isolation and may therefore be less resilient to novel threats, such as non-native species and the pathogens that may accompany them [79,80]. Moreover, more than half of the world’s poorest countries fall within biodiversity hotspots and rely on nature-tourism income [37]. The introduction and subsequent impacts of non-native species into these areas could therefore have serious economic, as well as ecological, ramifications [36,37]. However, the tourist income generated in these areas could also provide a source of revenue to fund management initiatives to prevent and mitigate the impacts of INNS [81]. For example, tourism funds up to 64% of global conservation measures for some bird species, measures which include the removal of INNS from critical habitat [81]. Such investment can in turn increase potential for eco-tourism in the long term [82].

Reducing unintentional introductions through the tourism pathway will require effective prediction, surveillance, awareness-raising and control [83], and will rely on international cooperation [10]. Awareness raising initiatives have already been developed to improve the biosecurity practices of recreational water users [59], hikers [84] and airline passengers [85] and have resulted in compliance by 71% of water users in New Zealand [86]. Minimum impact codes of conduct for visitors to national parks and protected areas have been proposed [24,87], and recently established in Europe [88], as a way of reducing non-native plant introduction by hikers and horse riders, as well as visitors taking part in recreational activities (e.g. motor boating, diving, snorkelling) in marine reserves [28]. A pan-European code of conduct for recreational boating is currently being drafted [89]while inspections of tourist footwear and luggage on arrival to pristine sites such as Antarctica have been suggested as another method a way to substantially reduced propagule loads [90]. However, many of these initiatives will need to be reinforced by legislation in order to be adopted [9]. On a larger scale, disinfection protocols have been implemented for inter-island aeroplanes and boats in the Galapagos Islands where the mosquito *Culex quinquefasciatus* had frequently been transported in aircraft [79,91].

INNS are a major threat to global biodiversity. In order to meet international conservation commitments, countries are obliged to identify and manage pathways for the spread of INNS species [92]. This meta-analysis has demonstrated that tourism and recreation can be significant pathways for the introduction of non-native species across all ecosystems. As the nature-tourism and outdoor recreation sector continues to grow [36], so too will the need for effective and long-lasting biosecurity interventions.

## Supporting Information

S1 PRISMA Checklist PRISMA Checklist. (DOC)

S1 Fig Publication bias evaluation. Normal quantile plots of the standardised effect sizes (*Hedges g*) against normal quantiles for the studies included in the meta-analysis to assess the responses of non-native species A) richness and B) abundance in sites where tourism/recreation took place. All points fall within the 95% confidence intervals indicating that the data are normally distributed.
(DOCX)

S2 Fig Forest plots. Plots depict the effect sizes of each individual study included in the meta-analysis.
(DOCX)

S1 Table Details of the articles rejected from the systematic review at full text stage along with reasoning. (XLSX)

S2 Table Characteristics of the 37 studies included in the final meta-analysis. (XLSX)
