# Supplementary material for: The Role of Tourism and Recreation in the Spread of Non-Native Species: A Systematic Review and Meta-Analysis
Source: PLoS One. 2015 Oct 20;10(10):e0140833. doi: 10.1371/journal.pone.0140833 (PMC4618285; doi:10.1371/journal.pone.0140833)
Supplement: S1 Fig — Normal quantile plots of the standardised effect sizes (Hedges g) against normal quantiles for the studies included in the meta-analysis to assess the responses of non-native species A) richness and B) abundance in sites where tourism/recreation took place. All points fall within the 95% confidence intervals indicating that the data are normally distributed. (DOCX) [file pone.0140833.s002.docx]

| 1. Studies investigating the effect of recreation of non-native species abundance   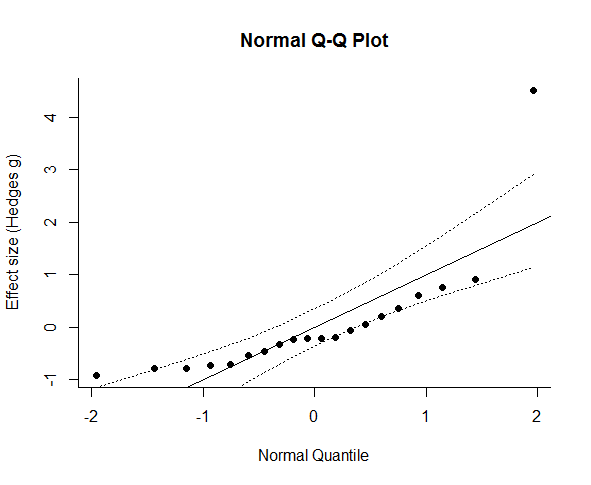 |
| --- |
| 1. Studies investigating the effect of recreation on non-native species richness   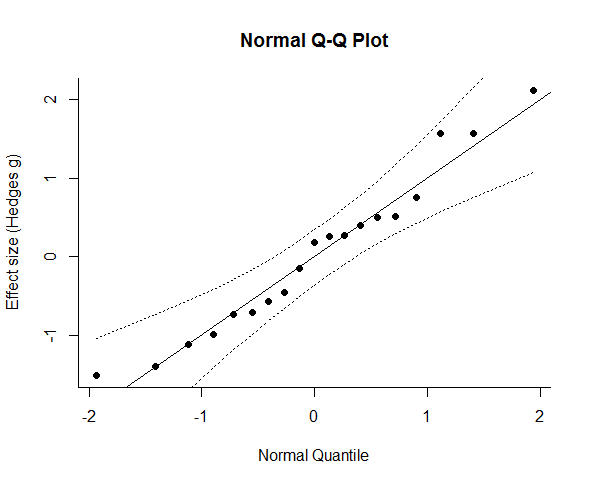 |
| Figure S1. Normal quantile plots of the standardised effect sizes (*Hedges g*) against normal quantiles for the studies included in the meta-analysis to assess the responses of non-native species A) abundance and B) richness in sites disturbed by tourism/recreation. All but one point fall within the 95% confidence intervals indicating that the data are normally distributed. |
