# Supplementary material for: The Role of Tourism and Recreation in the Spread of Non-Native Species: A Systematic Review and Meta-Analysis
Source: PLoS One. 2015 Oct 20;10(10):e0140833. doi: 10.1371/journal.pone.0140833 (PMC4618285; doi:10.1371/journal.pone.0140833)
Supplement: S2 Fig — Plots depict the effect sizes of each individual study included in the meta-analysis. (DOCX) [file pone.0140833.s003.docx]

| A)  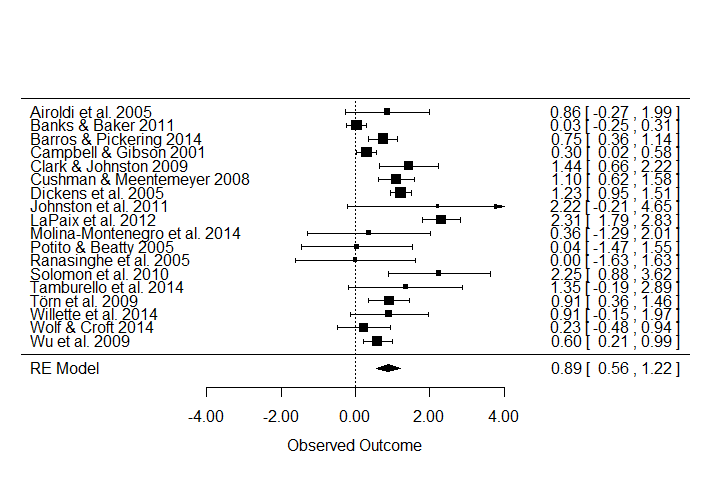 |
| --- |
| B)  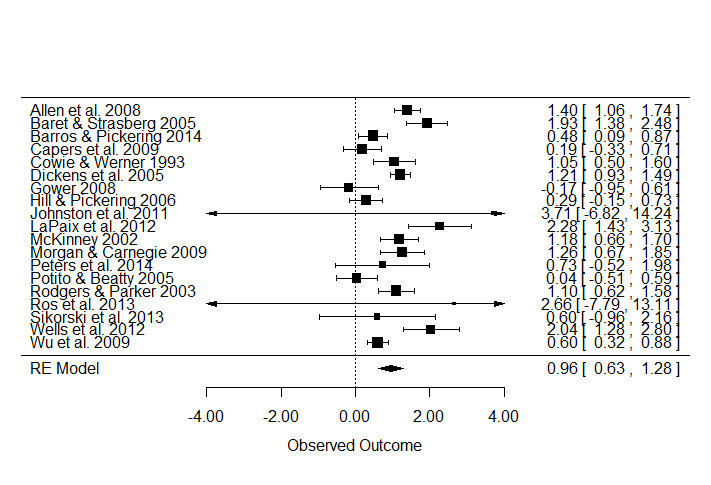 |
| Figure S2. Forest plots showing the effects of recreational activities on A) non-native species abundance and B) non-native species richness. The mean effect size (*Hedges g*) and 95% confidence intervals are provided for each study. |
